# Acoustic effects of non-transparent and transparent face coveringsa)

**DOI:** 10.1121/10.0003962

**Published:** 2021-04-02

**Authors:** Samuel R. Atcherson, B. Renee McDowell, Morgan P. Howard

**Affiliations:** Department of Audiology and Speech Pathology, University of Arkansas for Medical Sciences, Little Rock, Arkansas 72205, USA

## Abstract

The widespread use of face coverings during the COVID-19 pandemic has created communication challenges for many individuals, particularly for those who are deaf or hard of hearing and for those who must speak through masks in suboptimal conditions. This study includes some newer mask options as well as transparent masks to help those who depend on lipreading and other facial cues. The results corroborate earlier published results for non-transparent masks, but transparent options have greater attenuation, resonant peaks, and deflect sounds in ways that non-transparent masks do not. Although transparent face coverings have poorer acoustic performance, the presence of visual cues remains important for both verbal and non-verbal communication. Fortunately, there are creative solutions and technologies available to overcome audio and/or visual barriers caused by face coverings.

## I. INTRODUCTION

The novel coronavirus disease 2019 (COVID-19) brought many changes to daily life in 2020, including a recommendation by the Centers for Disease Control and Prevention (CDC) to wear face coverings over the mouth and nose (CDC, 2020). Consequently, many states and districts began to create mandates requiring citizens to wear masks in public places (Mendelson, 2020). This new norm has challenged access to clear speech communication. That is, conventional face coverings result in a noticeable reduction in the loudness and clarity of speech that can be perceived even by individuals with normal hearing sensitivity (Goldin *et al.*, 2020). Speech understanding is further reduced when there is competing background noise (Goldin *et al.*, 2020) and the effect of increased physical distance (Tucci, 2020; Wolfe *et al.*, 2020). Second, conventional face coverings not only deteriorate the quality of speech, but they also present a visual obstacle to facial cues and lipreading, especially for individuals who are deaf or hard of hearing (Mendel *et al.*, 2008; Atcherson *et al.*, 2017; Chodosh *et al.*, 2020; Atcherson *et al.*, 2020; Tucci, 2020; Eby *et al.*, 2020; Corey *et al.*, 2020). In addition to the loss of facial cues, the emotions conveyed by the speaker can also be disrupted (Tucci, 2020). Compounding the problem further are other health-related and communication influences, such as illness, cognitive decline, speech impairments, voice disorders, foreign accents, and speech dialects. Thus, face coverings create communication challenges in many social, educational, vocational, and health-related areas.

Early in the spring of 2020, Goldin *et al.* (2020) reported that surgical masks had an average attenuation of 3–4 dB, whereas N95 respirator masks could attenuate speech by as much as 12 dB. They articulated concerns about communication access in healthcare settings particularly for deaf or hard of hearing individuals and given the higher prevalence of hearing loss among men and in older adults who would be among those at-risk. When the CDC (2020) made its recommendations for widespread use of face coverings, Baltimore and Atcherson (2020) expressed concerns for clients and patients receiving speech-language and audiology services, yet another demographic group with a variety of speech, voice, language, and hearing communication disorders that would be impacted by greater mask use. High frequency attenuation by face coverings has the greatest impact on consonant speech sounds, which is also the region where hearing loss is present for the majority of deaf or hard of hearing individuals. Also, high frequency consonants are often masked by intense, low frequency sounds present in commonly experienced background noise. At around the same time, several groups began exploring the acoustic and behavioral impact of masks and shields with amplification and wireless assistive technology (Rudge *et al.*, 2020; Atcherson *et al.*, 2020; Corey *et al.*, 2020; Wolfe *et al.*, 2020). As schools, clinics, and businesses strive to remain open (or reopen), certain situations call for a combination of face coverings, the most common of which is a plastic face shield worn over a mask. In some cases, health professionals must wear an N95 respirator and surgical mask, both under a plastic face shield, while performing procedures at close proximity to patients (Bannerman, 2020). Most surprising, however, is the rise and interest in commercial and homemade transparent face covers to permit access to facial cues, which also provide a degree of protection from COVID-19. Although transparent masks perform poorly with sound transmission (Atcherson *et al.*, 2020), it has been demonstrated that the provision of both audio and visual cues can help all listeners: (1) learn and process non-verbal facial expressions specific to their language and culture (Elliot and Jacobs, 2013), (2) segment parts of speech better (Mitchel and Weiss, 2013), and (3) when there is background noise present (Atcherson *et al.*, 2017).

Thus, the purpose of this study was to expand on some of the previous work by Goldin *et al.* (2020), Atcherson *et al.* (2020), and Corey *et al.* (2020). First, none of the three studies examined the acoustic effects of some newer plastic shield-type options (e.g., Humanity Shield, ClearMask, and Moog face shield with apron) that provide greater visual access to the face, while also providing a measure of protection compared to a face shield alone. Second, and in advance of schools and universities opening that could impact learning, Atcherson *et al.* (2020) shared only preliminary data on a limited number of masks (at the time) with and without a face shield. Finally, it was of interest to explore the acoustic effects of masks at two fixed distances (3 and 6 ft) as certain professions require a closer working distance (e.g., frontline workers, speech-language pathologists, optometrists, hairdressers, etc.). As this study focuses principally on acoustic effects, the examination of amplification technology to overcome acoustic effects can be found elsewhere (Rudge *et al.*, 2020; Corey *et al.*, 2020; Wolfe *et al.*, 2020; NAL, 2020).

## II. METHODS

**FIG. 1.**
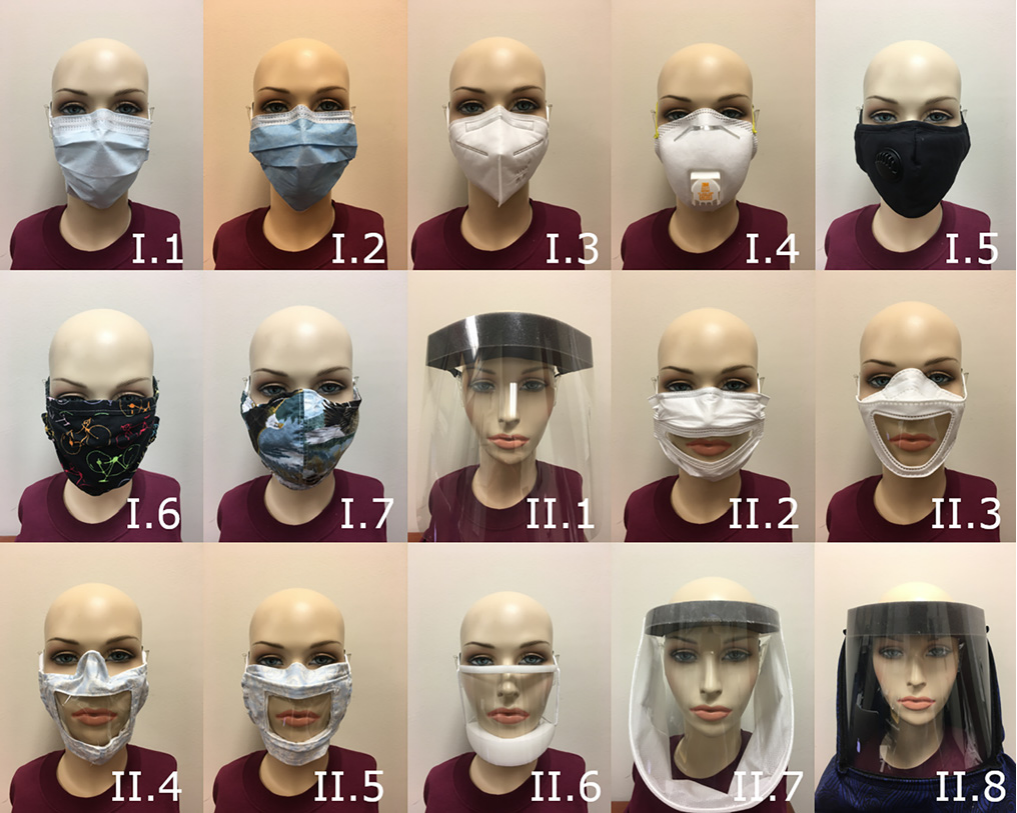
(Color online) Face coverings used and described in Tables I–III. The numbers correspond to those listed in Tables I and II.

Prior to the study, a variety of face coverings was procured for an acoustic study based on availability. These included two conventional surgical masks, two respirator masks (KN95, N95), one carbon filter mask (PM2.5), and two homemade cloth masks of different designs (one with a replaceable HEPA filter), two transparent masks (one under R&D), two homemade cloth masks with transparent windows, one transparent shield-type transparent mask (nose and mouth only), two plastic shields with coverings (one was homemade), and one generic plastic shield. These face coverings are shown in Fig. 1.

**FIG. 2.**
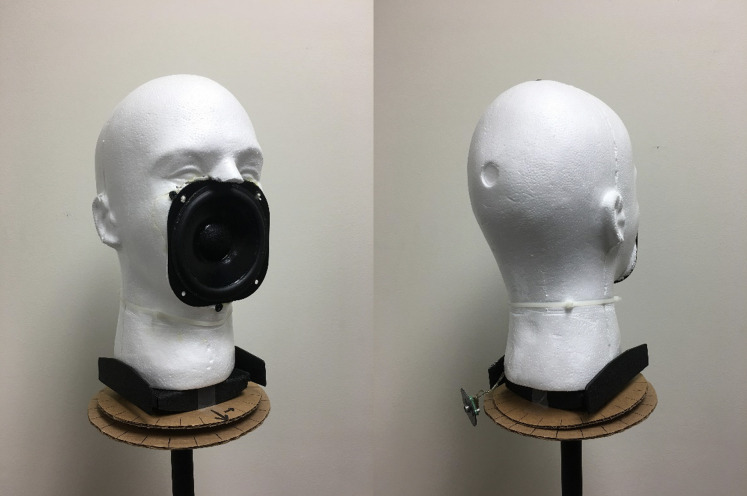
(Color online) White noise was presented through a head-shaped, custom mouth simulator: (1) with a “listener” microphone placed at distances of 3 and 6 ft to measure acoustic attenuation relative to the no mask condition, and (2) while rotating it in 15 degree increments with the “listener” microphone placed at a fixed distance of 6 ft.

A custom mouth simulator (Fig. 2) was fabricated as the “talker” using a styrofoam head (Bluelans B074RBHCFS) with midrange loudspeaker (Vifa C11WG-09) to present white noise from a compact disk player (Sony CDP-C245) and amplified (Realistic SA-150). The loudspeaker has a flat, 0 degree azimuth frequency response between 200 Hz and 6 kHz. The acoustic output through various face coverings was obtained at a distance of 3 and 6 ft using a digital recorder (Tascam DR-660) and a unidirectional, dynamic microphone (Shure SM48) as the “listener.” To characterize the directional effects of various transparent face coverings, the mouth simulator was turned in 15 degree increments, while the microphone remained fixed at a distance of 6 ft (see Corey *et al.*, 2020). All recordings took place inside of a double-walled, audiology test suite (Acoustic Systems RE 243) and the distance between the mouth simulator and “listener” microphone was equidistant between the floor and ceiling, and between one corner of the booth and its diagonal corner. Calibration of the white noise at the center of the booth (3 ft from mouth simulator) was maintained at 65 dB SPL. All recordings were obtained over 10 s of white noise to calculate the acoustic attenuation (in dB) for each face covering relative to the no mask condition and for direct comparison with Goldin *et al.* (2020). The root-mean-square (rms) levels were calculated for data points between 2 and 8 kHz, using the following formula for a given signal,

$$\begin{array}{c} x=\;\left\{x_{1},x_{2},\ldots ,x_{n}\right\},\;\text{the}\;\;\text{rms}\;\text{value},\;x_{\text{rms}},\;\text{is}\\ x_{\text{rms}}=\sqrt{\frac{x^{2}}{n}},\\ x_{\text{rms}}=\sqrt{\frac{1}{n}(x_{1}^{2}+x_{s}^{2}+\cdots +x_{n}^{2})}. \end{array}$$

## III. RESULTS AND DISCUSSION

### A. Acoustic attenuation of face covers

**FIG. 3.**
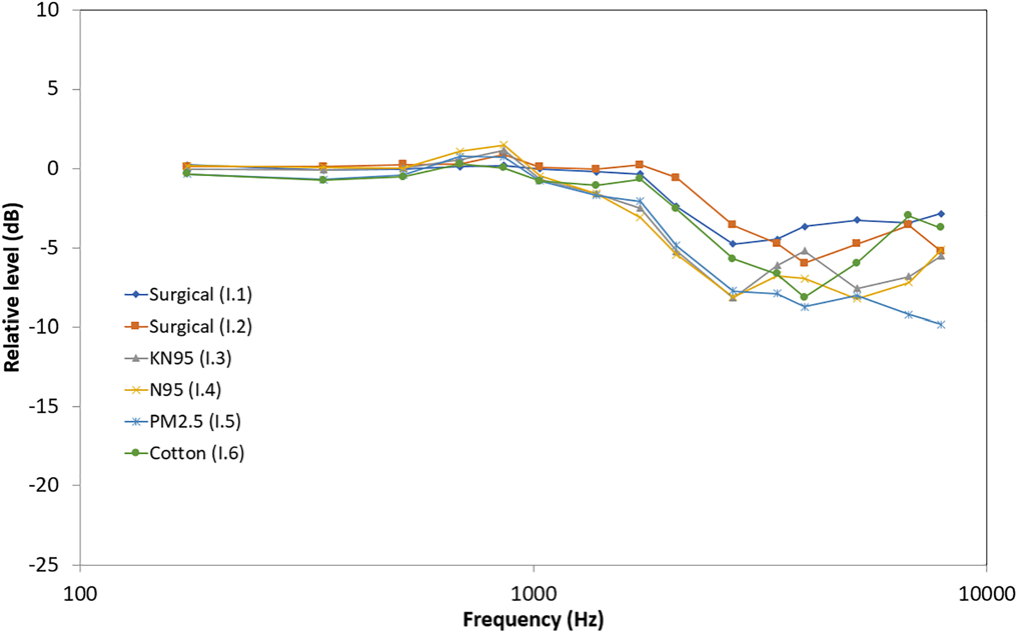
(Color online) Acoustic attenuation effects in dB for various non-transparent face covers placed on the head-shaped, custom mouth simulator relative to the no mask recording.

**FIG. 4.**
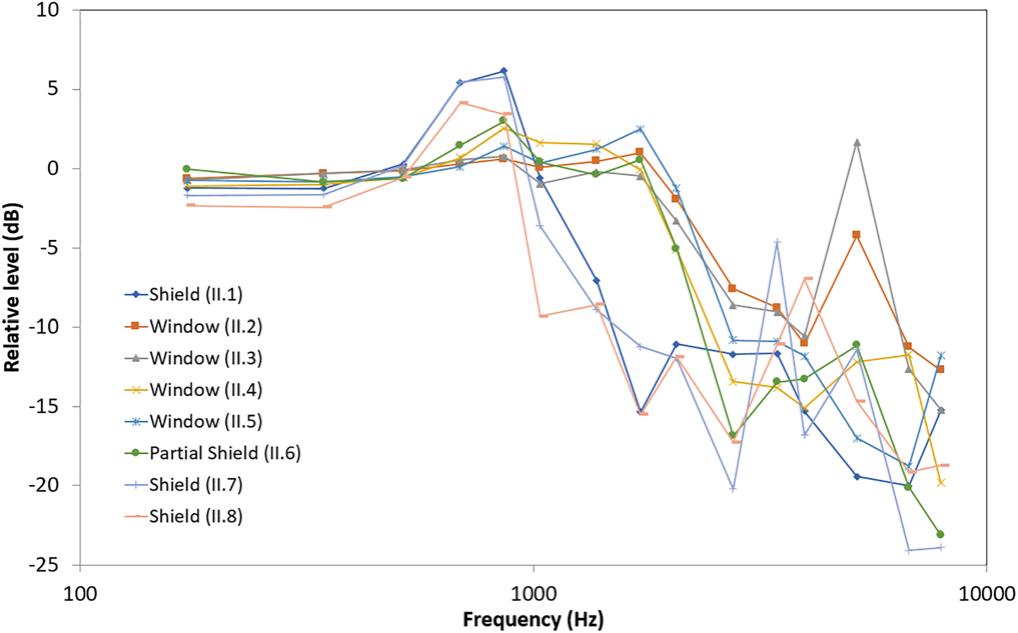
(Color online) Acoustic attenuation effects in dB for various transparent face covers placed on the head-shaped, custom mouth simulator relative to the no mask recording.

Figures 3 and 4 show the acoustic transfer functions measured at 6 ft (i.e., recommended social distance) for non-transparent and transparent face covers. Data points are plotted logarithmically relative to the no mask condition. For most face covers, there appears to be minimal attenuation and differences below 1 kHz with greater attenuation and variable differences in the higher frequencies, corroborating the well-established low-pass filtering effect. Tables I and II show at 3 and 6 ft the calculated rms level and acoustic attenuation relative to the no mask condition between 2 and 8 kHz for the non-transparent and transparent face covers, respectively. There is greater attenuation for the 6-ft distance on the order of 4 dB for Table I and 5 dB for Table II. However, the acoustic attenuation relative to the no mask condition is about 1–2 dB between the distances.

**TABLE I.** Non-transparent face covering acoustic attenuation rms results in dB between 2 and 8 kHz.

		Calculated rms		Attenuation re: No mask	
	Material	3 ft	6 ft	3 ft	6 ft
I.1	Polypropylene ASTM Level 2 (MediCom 2142)	16.2	21.9	3.6	3.5
I.2	Polypropylene ASTM Level 3 (DemeTECH)	16.8	22.6	4.2	4.2
I.3	KN95 respirator (Huixin GB-2626-2006)	18.8	24.6	6.3	6.3
I.4	N95 respirator (3 M 8511)	18.9	24.5	6.4	6.2
I.5	PM2.5 (Tworux)	20.9	26.4	8.4	8.0
I.6	Cloth (handmade)	17.9	23.4	5.4	5.1
I.7	Cloth with HEPA filter (handmade)	18.7	24.1	6.1	5.7

**TABLE II.** Transparent face covering acoustic attenuation rms results in dB between 2 and 8 kHz.

		Calculated rms attentuation		Attenuation re: No mask	
	Material	3 ft	6 ft	3 ft	6 ft
II.1	Plastic shield (generic)	28.0	33.5	15.5	15.2
II.2	The Communicator (Safe ‘N’ Clear)	21.5	26.9	9.0	8.5
II.3	FaceView (Jeanne Hahne)	22.4	27.8	9.8	9.4
II.4	Cotton/polyester blend and vinyl window 1 (handmade)	26.8	31.4	14.2	13.0
II.5	Cotton/polyester blend and vinyl window 2 (handmade)^a^	27.1	31.8	14.5	13.4
II.6	ClearMask (ClearMask LLC)	28.6	32.8	16.1	14.4
II.7	Humanity Shield (Rapid Response PPE)	29.7	34.9	17.2	16.5
II.8	Moog plastic shield with apron (handmade)^b^	28.1	32.1	15.5	13.7

^a^ See https://sewingseedsoflovestudio.com/products/ssol-smile-mask-pattern-free.

^b^ See https://amandarudge.files.wordpress.com/2020/08/appendix_the-effects-of-face-coverings-and-remote-microphone-technology-on-speech-perception-in-the-classroom.pdf.

The two surgical masks (I.1 and II.2) attenuated about 4 dB and the N95 respirator mask (I.4) attenuated about 6 dB similar to results reported by Goldin *et al.* (2020) and Corey *et al.* (2020). These results are slightly better than the maximum single data point attenuation reported by Atcherson *et al.* (2020). The KN95 respirator mask (I.3) attenuated about 6 dB, slightly poorer than the 4 dB reported by Corey *et al.* (2020). The reusable PM2.5 respirator mask with replacement carbon filter (I.5) had the greatest attenuation among non-transparent face covers at about 8 dB. The two cloth masks (I.6 and I.7) attenuated around 5–6 dB comparable to results reported by Corey *et al.* (2020). Surprisingly, the cloth mask with HEPA filter (adding a third layer) had only slightly more attenuation; however, both non-transparent cloth masks were made of two layers of plain cotton.

The transparent face covers varied widely in their effects with greater attenuation (between 9 and 17 dB) compared to the non-transparent face covers (cf. Tables I and II). The face shield (II.1) and shield-like types (II.6, II.7, and II.8) appear to amplify sounds in the 0.5–1 kHz range similar to the face shield results by Corey *et al.* (2020), and all transparent face covers have unique “resonance-like” peaks between 5 and 7 kHz similar to findings by Atcherson *et al.* (2020) and Corey *et al.* (2020). Not unexpected, full face shields performed poorly compared to the other non-transparent face covers, particularly in the 1–3 kHz range. Of the transparent options that cover only the nose and mouth, the Safe ‘N’ Clear (II.2) and FaceView (II.3) masks performed the best, and the partial shield ClearMask (II.4) performed the worst. For both listeners with and without hearing loss, transparent face covers provide visual access to the face (in part or in full), but they degrade high-frequency speech cues (Goldin *et al.*, 2020; Atcherson *et al.*, 2020; Corey *et al.*, 2020).

### B. Acoustic attenuation of face coverings with a standard face shield

Table III shows the acoustic attenuation for all nose and mouth face covers worn with and without a generic plastic face shield (to protect the eyes) relative to the no mask condition. An additional condition combining the face shield with the N95 respirator mask worn under a surgical mask is also listed (III.12). By and large, the addition of the face shield worn with one or more nose and mouth face covers results from a 10 to 16 dB greater attenuation for a combined attenuation that ranges from 18 to 25 dB. Atcherson *et al.* (2020) reported slightly poorer results using the maximum single data point between 2 and 8 kHz with attenuations of 8 to 20 dB contributed by the face shield and combined mask and face shield attenuation ranging from 20 to 29 dB.

**TABLE. III:** Combined face coverings and plastic shield acoustic attenuation RMS results in dB between 2 and 8 kHz relative to no mask condition.

		No Shield		With Shield	
	Material	3 ft	6 ft	3 ft	6 ft
III.1	Polypropylene ASTM Level 2 (MediCom 2142)	3.6	3.5	19.5	18.1
III.2	Polypropylene ASTM Level 3 (DemeTECH)	4.2	4.2	19.9	18.0
III.3	KN95 respirator (Huixin GB-2626-2006)	6.3	6.3	20.0	19.5
III.4	N95 respirator (3 M 8511)	6.4	6.2	23.0	21.4
III.5	PM2.5 (Tworux)	8.4	8.0	23.5	22.4
III.6	Cloth (handmade)	5.4	5.1	20.5	18.3
III.7	Cloth with HEPA filter (handmade)	6.1	5.7	22.1	20.2
III.8	The Communicator (Safe ‘N’ Clear)	9.0	8.5	22.5	22.3
III.9	FaceView (Jeanne Hahne)	9.8	9.4	23.6	22.2
III.10	Cotton/polyester blend and vinyl window 1 (handmade)	14.2	13.0	25.0	23.6
III.11	Cotton/polyester blend and vinyl window 2 (handmade)^a^	14.5	13.4	25.0	24.6
III.12	III.1 (Polypropylene 1) and III.4 (N95) doubled up	9.3	9.0	25.7	24.9

^a^ See https://sewingseedsoflovestudio.com/products/ssol-smile-mask-pattern-free.

### C. Directional effects of transparent face coverings

**FIG. 5.**
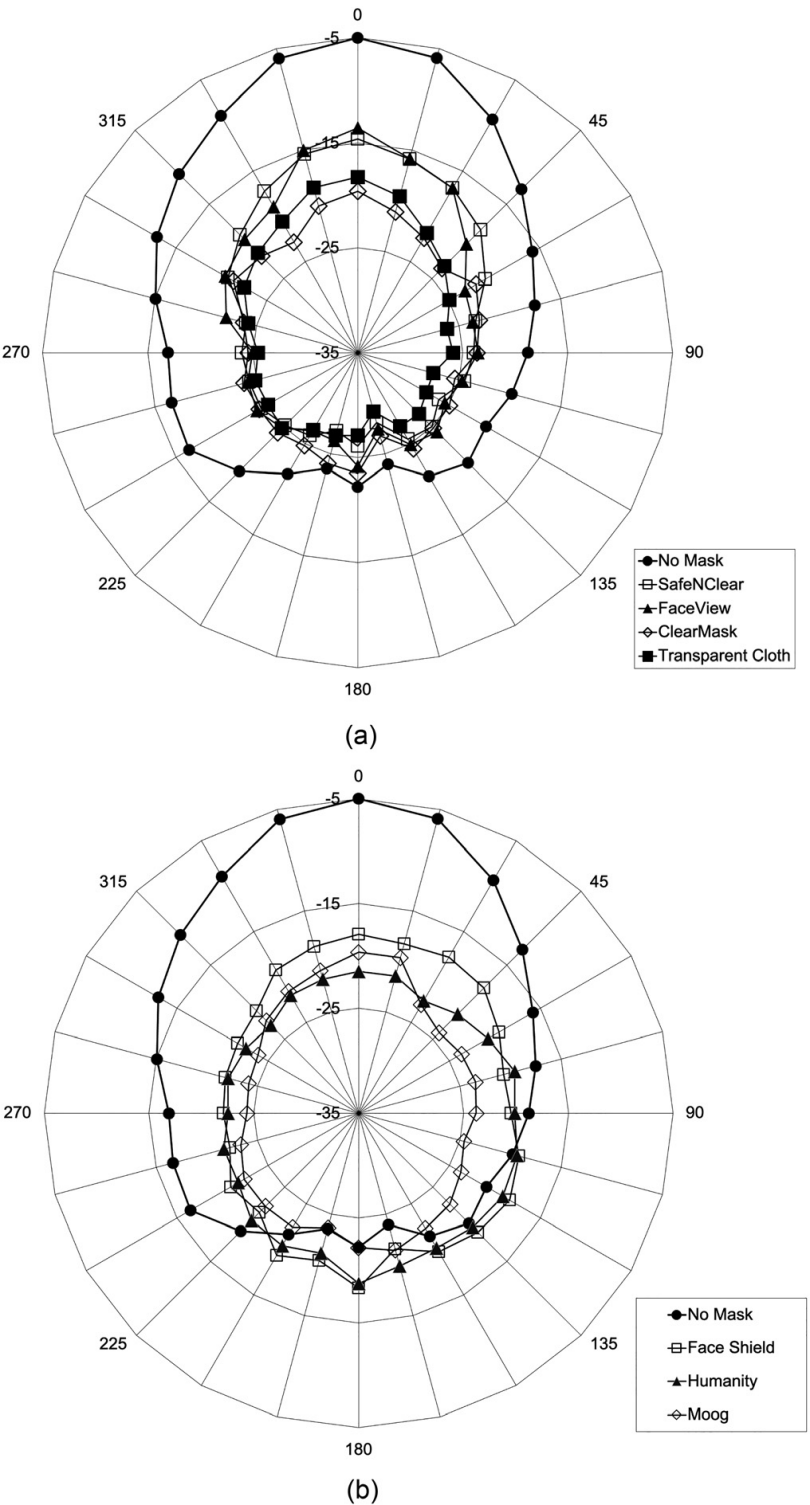
Polar plots of directional sound energy for various transparent face coverings relative to no mask as the head-shaped, custom mouth simulator was rotated in 15 degree increments (right, mouth/nose transparent coverings; left, full face transparent coverings). The nose of the mouth simulator is located at 0 degrees and the outer ring represents the highest intensity.

Figure 5 shows the directional effects of transparent face coverings as a function of angle for the head-shaped, custom mouth simulator with the “listener” microphone 6 ft away. For each of the 15 degree rotations, the rms level for data points between 2 and 8 kHz was plotted. In general, all transparent face coverings restrict sound transmission from all angles relative to no mask. The nose/mouth types appear to have less restriction towards the front and greater attenuation on the sides and back compared to full face types. The shield-like types that cover most, if not all, parts of the face [e.g., face shield (II.1), ClearMask (II.6), and Humanity Shield (II.7)] appear to deflect and amplify sounds to the side and back relative to the non-shield types. The transparent cloth mask (II.4) and Moog shield with apron (II.8) are among the two most restrictive types due to poorer sound transmission through two layers of cotton. Because an audiology test booth was used for all recordings, the directional plots are likely influenced by reflective surfaces at the corners of the booth. For all transparent face covers, there also appear to be relationships among variables such as the size of the window/shield, the distance between the window/shield and the mouth, and the relative fit, which will vary from person to person and how the face cover is worn.

## IV. CONCLUSIONS

The results of this study are in agreement with the results by Goldin *et al.* (2020) and Corey *et al.* (2020), and also provide previously unreported information about transparent options beyond commercial and handmade masks with windows and plastic face shields. This study confirms that face covers of all types attenuate high frequency sounds above 1 kHz, but transparent options have greater attenuation compared to those that are non-transparent.

Herein lies an interesting situation: All face coverings can negatively impact speech understanding by acoustic attenuation alone when used independently or in combinations. While transparent masks and shields can overcome some of the barriers to speech understanding by the provision of facial and emotional cues, they have, on average, greater attenuation compared to their non-transparent counterparts. When examining the transparent options more closely, the full and partial face shields have greater forward attenuation compared to those that only cover the nose and mouth, and they can deflect sound towards the sides and back. Additionally, when the face shield is combined with one or more masks, the addition of the shield can add greater attenuation of about 10–16 dB and a total combined attenuation of 18–25 dB. Regardless of the face covering options available or employed, the results of this study (and of others) clearly demonstrate the need for supplemental solutions to overcome barriers to speech communication.
